# Familial cleft tongue caused by a unique translation initiation codon variant in *TP63*

**DOI:** 10.1038/s41431-021-00967-x

**Published:** 2021-10-11

**Authors:** Julia Schmidt, Gudrun Schreiber, Janine Altmüller, Holger Thiele, Peter Nürnberg, Yun Li, Silke Kaulfuß, Rudolf Funke, Bernd Wilken, Gökhan Yigit, Bernd Wollnik

**Affiliations:** 1 Institute of Human Genetics, University Medical Center Göttingen, Göttingen, Germany; 2 Department of Pediatric Neurology, Klinikum Kassel, Kassel, Germany; 3 Cologne Center for Genomics (CCG), University of Cologne, Faculty of Medicine, University Hospital Cologne, Cologne, Germany; 4 Center for Molecular Medicine Cologne (CMMC), University of Cologne, Faculty of Medicine, University Hospital Cologne, Cologne, Germany; 5 Berlin Institute of Health at Charité, Core Facility Genomics, Berlin, Germany; 6 Max Delbrück Center for Molecular Medicine in the Helmholtz Association, Berlin, Germany; 7 Cluster of Excellence “Multiscale Bioimaging: from Molecular Machines to Networks of Excitable Cells” (MBExC), University of Göttingen, Göttingen, Germany

**Keywords:** Disease genetics, Genetics research, Development

## Abstract

Variants in transcription factor p63 have been linked to several autosomal dominantly inherited malformation syndromes. These disorders show overlapping phenotypic characteristics with various combinations of the following features: ectodermal dysplasia, split-hand/foot malformation/syndactyly, lacrimal duct obstruction, hypoplastic breasts and/or nipples, ankyloblepharon filiforme adnatum, hypospadias and cleft lip/palate. We describe a family with six individuals presenting with a striking novel phenotype characterized by a furrowed or cleft tongue, a narrow face, reddish hair, freckles and various foot deformities. Whole-exome sequencing (WES) identified a novel heterozygous variant, c.3G>T, in *TP63* affecting the translation initiation codon (p.1Met?). Sanger sequencing confirmed dominant inheritance of this unique variant in all six affected family members. In summary, our findings indicate that heterozygous variants in *TP63* affecting the first translation initiation codon result in a novel phenotype dominated by a cleft tongue, expanding the complex genotypic and phenotypic spectrum of *TP63*-associated disorders.

## Introduction

Heterozygous variants in transcription factor p63 encoded by *TP63* [MIM 603273] have been linked to several autosomal dominantly inherited malformation syndromes including ankyloblepharon-ectodermal defects-cleft lip/palate syndrome (AEC [MIM 106260]), acro-dermo-ungual-lacrimal-tooth syndrome (ADULT [MIM 103285]), ectrodactyly, ectodermal dysplasia, cleft lip/palate syndrome 3 (EEC3 [MIM 604292]), limb-mammary syndrome (LMS [MIM 603543]), split-hand/foot malformation type 4 (SHFM4 [MIM 605289]) and isolated orofacial cleft 8 (OFC8 [MIM 618149]) [1]. *TP63*-related disorders have overlapping phenotypic characteristics with various combinations of the following features: ectodermal dysplasia (subjective hypohidrosis, nail dysplasia, sparse hair, skin erosions especially on the scalp associated with areas of scarring and alopecia, hypopigmentation, trismus, excessive freckling, tooth abnormalities), split-hand/foot malformation/syndactyly, lacrimal duct obstruction, hypoplastic breasts and/or nipples, ankyloblepharon filiforme adnatum, hypospadias and cleft lip/palate [1, 2].

**Fig. 1:**
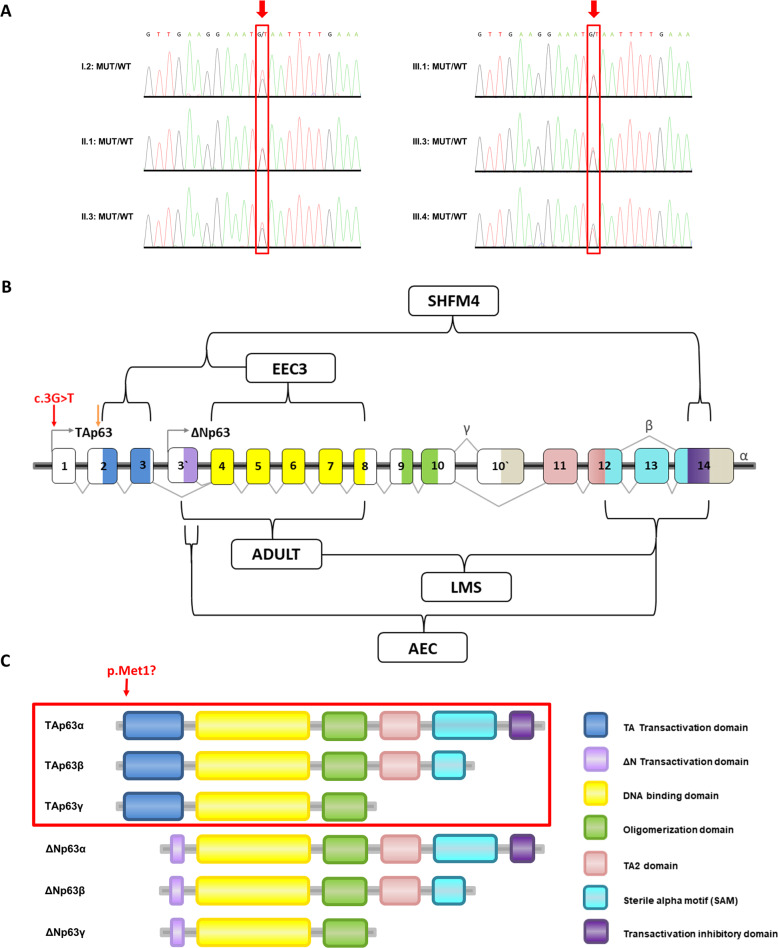
Overview of the identified translation initiation codon variant in *TP63* on genomic and protein level. **A** Chromatograms of the identified *TP63* variant in six affected family members (I.2, II.1, II.3, III.1, III.3 and III.4: c.3G>T; p.Met1?). **B** Schematic diagram of the human *TP63* gene structure. Alternative promoter use produces TA (transactivation) and N-terminally truncated (ΔN) isoforms, and alternative splicing produces C-terminal variants (α, β, γ). Colors within exons correspond to the different functional domains. Red arrow indicates the *TP63* variant identified within this study. Orange arrow indicates the most likely alternatively used start codon at position 40, resulting in a shortened version of the TAp63 isoforms. Different *TP63*-associated disorders (ankyloblepharon-ectodermal defects-cleft lip/palate syndrome (AEC); acro-dermo-ungual-lacrimal-tooth syndrome (ADULT); ectrodactyly, ectodermal dysplasia, cleft lip/palate syndrome 3 (EEC3); limb-mammary syndrome (LMS); split-hand/foot malformation type 4 (SHFM4)) and the typical location of their variant are indicated by black brackets. **C** Comparison of the six major isoforms encoded by TP63. Isoforms in the red box are potentially affected by the TP63 variant p.Met1?. Colors correspond to the different functional domains.

*TP63* is located on 3q28 and comprises 16 different exons [3, 4]. It is a member of the large p53 family of transcription factors and a key regulator in epidermal development and differentiation [5]. Additionally, p63 has been linked to divers biological processes, e.g., differentiation, proliferation, adhesion, stem cell maintenance, apoptosis and aging [6, 7]. The wide spectrum of cellular functions might be explained by the large number of p63 isoforms identified yet. Two different transcription start sites lead to two main classes of transcripts: TAp63 and ΔNp63. TAp63 contains three TA-specific exons, exon 1, 2 and 3, and encodes a transactivation domain (Fig. 1). The shorter isoform variant, ΔNp63, uses an independent promotor in exon 3’ resulting in a truncated transactivating domain [6, 8, 9]. In addition, alternative splicing at the 3ʹ end of both the TAp63 and ΔNp63 transcripts generates at least three different C-terminal variants (α, β, and γ) [8, 10]. The longest 3′ splice variant (including the exons 11–14) is the α isoform, which comprises a sterile alpha-motif (SAM) involved in protein–protein interactions and a transactivation inhibitory domain (TID) [10–13]. Two further variants, β (which lacks exon 13) and γ (that does not contain the exons 11–14 but has a γ-specific exon 10′), do not comprise the SAM and TID domains [14, 15]. All isoforms contain the DNA-binding domain and oligomerization domain (OD) [5, 8, 10, 16]. A wide spectrum of different heterozygous germline variants in *TP63* have been identified as causative of the syndromes and malformations stated above. According to the literature (HGMD Database Professional 2020.4), most of the reported disease-causing variants (110 of 153) are missense variants. Variants associated with particular disorders usually cluster in specific functional domains, indicating a genotype-phenotype association [2, 4, 17]. For instance, variants causing the EEC3 syndrome are generally located in the DNA-binding domain, whereas variants leading to the AEC phenotype are regularly found either in the SAM and transactivation inhibitory domains or in the transactivating domain of the ΔNp63 isoforms (Fig. 1) [2, 5, 18, 19]. These findings suggest that there might be specific molecular mechanisms for each *TP63*-related condition.

Here, we describe a unique translation initiation codon variant in *TP63* identified in a family with a striking novel phenotype. The variant, c.3G>T, affects the first transcription start site of p63 and is predicted to lead to impaired function of the TAp63 isoforms. All six patients carrying this variant show a cleft tongue.

## Clinical report

**Fig. 2:**
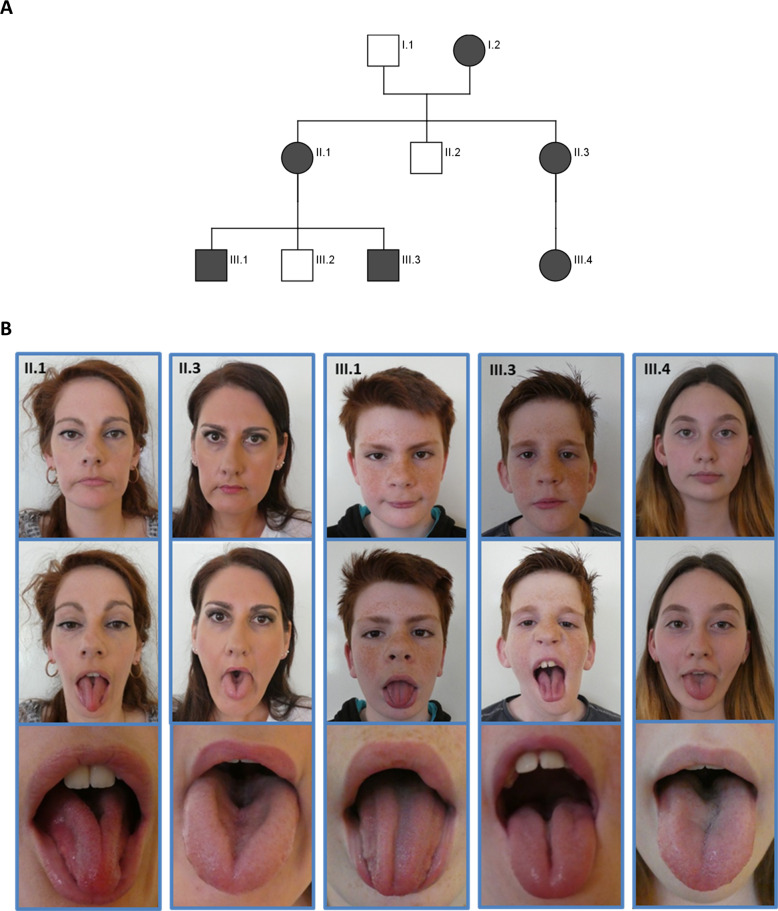
Pedigree and clinical characteristics of individuals carrying the heterozygous c.3G>A variant in *TP63*. **A** Family pedigree, unfilled shapes denote healthy individuals, filled shapes indicate those family members who are clinically affected. **B** Clinical characteristics of patient II.1, II.3, III.1, III.3 and III.4. Facial features included a long narrow face (HP:0000275), hypotrophic jaw muscles (HP:0045037), reddish hair (HP:0002297), freckels (HP:0001480) and a midline furrow of the tongue (HP:0000221).

We describe six patients with cleft tongue (HP:0000221) who were referred to our Institute of Human Genetics for clinical and diagnostic evaluation. The cleft tongue (HP:0000221) was already present at birth and did not change over time. Their sense of taste was not affected and no signs of submucous cleft palate were observed. The patients reported no difficulty in swallowing. The clinical characteristics of the patients described in the present report are summarized in Table 1. The family pedigree is illustrated in Fig. 2A and is consistent with autosomal dominant inheritance. The corresponding images are shown in Fig. 2B. The main clinical features are summarized and compared to the typical clinical findings of other *TP63*-associated disorders in Table 2.

**Table 1:** Clinical data of patients described in the present report.

Detailed clinical data						
ID	III.1	III.3	II.1	I.2	III.4	II.3
*TP63*/p63 variant	c.3G>T; (p.Met1?)	c.3G>T; (p.Met1?)	c.3G>T; (p.Met1?)	c.3G>T; (p.Met1?)	c.3G>T; (p.Met1?)	c.3G>T; (p.Met1?)
Zygosity	Heterozygous	Heterozygous	Heterozygous	Heterozygous	Heterozygous	Heterozygous
Gender	Male	Male	Female	Female	Female	Female
**Growth parameters and development**						
Birth (gestational age)	39 ws	40 ws	N/A	N/A	38 ws	42 ws
Birth length	53 cm (−0.5 SD)	56 cm (1.5 SD)	N/A	N/A	52 cm (0.7 SD)	51 cm (−0.7 SD)
Birth weight	3350 g (−0.3 SD)	3630 g (median)	N/A	N/A	3500 g (0.8 SD)	3300 g (−0.8 SD)
Head circumference at birth	35 cm (−0,2 SD)	34 cm (0.3 SD)	N/A	N/A	34 cm (−0.2 SD)	N/A
Age at examination	12 y 4 m	10 y 6 m	39 y	N/A	12 y	37 y
Height	167 cm (1.6 SD)	145 cm (0.2 SD)	171 cm (0.5 SD)	N/A	170 cm (2.1 SD)	166 cm (−0.4 SD)
Weight	66 kg (1.8 SD)	37.9 kg (0.2 SD)	N/A	N/A	54.1 kg (+1.1 SD)	N/A
Head circumference	55 cm (0.4 SD)	55 cm (0.9 SD)	56 cm (0.5 SD)	N/A	54 cm (0.4 SD)	55 cm (−0.6 SD)
Mental development	Normal	Normal	Normal	N/A	Normal	Normal
Hypotonia (HP:0001252)	+	+	+	N/A	(+)	++
Motor delay (HP:0001270)	Mild	Mild	−	N/A	Mild	+
Able to walk	1 y 3 m	1 y 4 m	N/A	N/A	1 y 3 m	2 y 6 m
**Clinical manifestations**						
Cleft tongue (HP:0000221)	+	+	+	+	+	+
Reddish hair (HP:0002297)	+	+	+	N/A	+	+
Freckles (HP:0001480)	+	+	+	N/A	+	+
Narrow face (HP:0000275)	+	+	+	N/A	+	+
Foot deformity (HP:0001760)	Talipes equinovarus (HP:0001762)	Pigeon toes (HP:0001760)	−	Pes equinus (HP:0001762)	Flat valgus feet (HP:0001763, HP:0008081)	Talipes equinovarus (HP:0001762)
**Other findings**						
Additional disorders		Langerhans’ cell histiocytosis at the age of 2 y and 4 m	−	Myopathy (HP:0003198)	Left-sided hearing loss (HP:0000365) (after Streptococcus pneumonia meningitis)	Myopathy (HP:0003198)

*+* present, *−* absent, *N/A* not available, *y* year, *m* month, *ws* weeks, *SD* standard deviation, *cm* centimeters, *HP* Human Phenotype Ontology.

**Table 2:** Summary of the clinical findings in our patients with the main clinical feature of cleft tongue (CT) (HP:0000221) compared to the typical clinical findings of different *TP63*-associated disorders: ankyloblepharon-ectodermal defects-cleft lip/palate syndrome (AEC); acro-dermo-ungual-lacrimal-tooth syndrome (ADULT); ectrodactyly, ectodermal dysplasia, cleft lip/palate syndrome 3 (EEC3); limb-mammary syndrome (LMS); split-hand/foot malformation type 4 (SHFM4) and isolated orofacial cleft 8 (OFC8) [1]. Human Phenotype Ontology (HPO) [40].

Feature		*TP63*-related disorder						
	HPO	CT	AEC	ADULT	EEC3	LMS	SHFM4	OFC8
Ankyloblepharon filiforme adnatum	HP:0009755		+					
Ectodermal dysplasia:	HP:0000968		+	+	+		rare	
Hypohidrosis (mostly subjective)	HP:0000966		+	+		+		
Nail dysplasia	HP:0002164		+	+	mild	+		
Sparse hair	HP:0008070		+	+	+			
Tooth abnormalities	HP:0000164		+	+	+	+		
Cleft lip/palate	HP:0000202		+		+	+		+
Cleft/furrowed tongue	HP:0000221	+						
Split-hand/foot malformation/syndactyly	HP:0002813		+	+	+	+	+	
Lacrimal duct obstruction	HP:0000579		+	+	+	+		
Dermal erosions	HP:0200041		+					
Hypopigmentation	HP:0007513		+	+	+			
Hypospadias	HP:0000047		+		+			
Trismus	HP:0000211		+					
Freckling	HP:0001480	+		+				
Hypoplastic breasts	HP:0010311			+				
Hypoplastic nipples	HP:0006709			+				

### Patient 1

The boy (III.1 in Fig. 2A) was born spontaneously after 39 weeks of gestation. Birth weight was 3350 g (−0.3 SD), length 53 cm (−0.5 SD) and occipitofrontal head circumference (OFC) 35 cm (−0.2 SD). Apgar scores were 10 at 5 and 10 min, respectively. Due to bilateral talipes equinovarus (HP:0001762) he required a series of castings followed by night splinting. His psychomotor development was slightly delayed. He walked independently at the age of 15 months and started to speak first words at the age of 2 years. At clinical evaluation at 12 years and 4 months, height was 167 cm (1.6 SD), weight 65.9 kg (1.8 SD), and OFC was 55.2 cm (0.4 SD). He showed reddish hair (HP:0002297), freckles (HP:0001480), and a narrow face (HP:0000275). His tongue showed a deep single median furrow looking like a cleft tongue (HP:0000221). Serum creatine kinase levels were unremarkable.

### Patient 2

III.3 in Fig. 2A was born at term. His birth weight was 3630 g (median), length 56 cm (1.5 SD) and OFC 36 cm (0.3 SD). Apgar scores were 10 at 5 and 10 min. Pigeon toes (HP:0001760) were noted shortly after delivery. His development was slightly delayed. He walked independently at the age of 16 months and started to speak first words at the age of 20 months. He received speech- and physiotherapy. At the age of 2 years and 4 months, he was diagnosed with Langerhans’ cell histiocytosis, which could be treated successfully with radiation. At 10 years and 6 months, his height was 145 cm (0.2 SD), weight 37.9 kg (0.3 SD), and OFC was 55 cm (0.9 SD). He showed the following facial features: narrow face (HP:0000275), reddish hair (HP:0002297), freckles (HP:0001480), and a median furrowed tongue/cleft tongue (HP:0000221). Serum creatine kinase levels were unremarkable.

### Patient 3

The mother of patients III.1 and III.3 (II.1) showed similar facial features as her affected sons including a narrow face (HP:0000275), reddish hair (HP:0002297), freckles (HP:0001480) and a median furrowed tongue/cleft tongue (HP:0000221). She reported a mild muscular hypotonia (HP:0001252) of her legs. Her height was 171.5 cm (0.5 SD), and OFC was 56 cm (−0.5 SD).

### Patient 4

Patient I.2 showed also a median furrowed tongue/cleft tongue (HP:0000221). She had a pointed foot as a child and reported muscular hypotonia (HP:0001252). She reported that her already deceased mother had similar symptoms, too.

### Patient 5

The girl (III.4 in Fig. 2A) was born after 38 weeks of gestation by Caesarean section. Her birth weight was 3500 g (0.8 SD), length was 52 cm (0.75 SD) and OFC 34 cm (−0.2 SD). Apgar scores were 10 at 5 and 10 min, respectively. She received physiotherapy due to muscular hypotonia (HP:0001252) and uses orthopedic insoles due to flat valgus foot. Otherwise, her psychomotor development was normal. She walked independently at the age of 15 months and started to speak first words at the age of 1 year. At clinical evaluation at 12 years and 4 months, her height was 167.2 cm (1.6 SD), weight 65.9 kg (1.8 SD), and OFC was 55.2 cm (0.4 SD). She showed the following characteristic facial features: narrow face (HP:0000275), reddish hair (HP:0002297), freckles (HP:0001480), and a median furrowed tongue/cleft tongue (HP:0000221).

### Patient 6

Patient II.3 showed a narrow and hypotonic facies (HP:0000275), reddish hair (HP:0002297), and a median deeply grooved tongue (HP:0000221). Bilateral talipes equinovarus (HP:0001762) were noted shortly after birth. She had surgical extension of the Achilles tendon at the age of three months and required a series of castings. Congenital hip dysplasia (HP:0001385) was treated with Pavlik Harness. Her motor development was delayed. She walked independently at the age of 2 years and 6 months. She reported muscular hypotonia (HP:0001252) of her legs. Her height was 165.8 cm (−0.4 SD) and OFC was 54.5 cm (−0.7 SD). Earlier genetic testing for facioscapulohumeral muscular dystrophy (FSHD), Emery–Dreifuss muscular dystrophy, Hauptmann–Thannhauser muscular dystrophy and Charcot-Marie-Tooth disease was unremarkable.

## Molecular studies and results

Written informed consent was obtained from all participants or their legal representatives prior to the participation in the study, and DNA from participating family members was extracted from peripheral blood lymphocytes by standard extraction procedures. The study was approved by the Ethics Committee of University Medical Center Göttingen (approval number 3/2/16) and performed in accordance with the Declaration of Helsinki protocols. We performed whole-exome sequencing (WES) on DNA extracted from blood of the patients III.1, III.2 and III.4, using the Agilent SureSelectXT Human All Exon V7 enrichment kit on an Illumina HiSeq4000 sequencer. The exome and genome analysis pipeline “Varbank 2.0” (https://varbank.ccg.uni-koeln.de/varbank2) of the Cologne Center for Genomics (CCG, University of Cologne, Germany) was used to analyze the exome data using the following filter criteria: coverage of more than 6 reads, a minimum quality score of 10, and a minor allele frequency (MAF) < 1.0% in the gnomAD (https://gnomad.broadinstitute.org) database. We obtained a mean coverage of 92–99 and 95.7%–96.2% of targets were covered more than 10×. The following databases were used to obtain gene information: National Center for Biotechnology Information (NCBI; https://www.ncbi.nlm.nih.gov), Ensembl Genome Server (http://www.ensembl.org), UCSC Genome Bioinformatics (http://genome-euro.ucsc.edu) and Genome Aggregation Database (gnomAD; http://gnomad.broadinstitute.org). We focused on heterozygous variants present in all three affected individuals consistent with a dominant transmission in line with the family history. We identified a heterozygous variant in *TP63* (RefSeq NM_003722.5) not present in any current database of human genetic variations including the gnomAD (https://gnomad.broadinstitute.org) database (last access date 10/02/2021, [20]) and predicted to have a severe impact on protein function. This heterozygous missense variant, c.3G>T, was located in exon 1 of the *TP63* gene (Fig. 1) and affects the first transcription start site of p63, leading to impaired TAp63 isoforms (p.Met1?). The variant was predicted as disease-causing by MutationTaster (http://www.mutationtaster.org), damaging by SIFT (https://sift.bii.a‐star.edu.sg), probably damaging by PolyPhen‐2 (http://genetics.bwh.harvard.edu/pph2), and has a CADD (https://cadd.gs.washington.edu) score of 25.6, indicating deleteriousness of this variant. According to NetStart 1.0 (score of 0.599; http://www.cbs.dtu.dk/services/NetStart/), TIS Miner (score of 0.791; http://dnafsminer.bic.nus.edu.sg/Tis.html), ATGpr (score of 0.48; https://atgpr.dbcls.jp/cgi-bin/atgpr.cgi) and the ORF finder (https://www.ncbi.nlm.nih.gov/orffinder/) the most likely alternatively used start codon is the next downstream AUG codon at position 40, resulting in a shortened version of the isoform originally annotated as TAp63 (Fig. 1B). Sanger sequencing was used to validate the WES data and to prove that the other affected family members, II.1, II.3 and I.2, do carry the heterozygous variant as well (Fig. 2A). No additional patient material was available for detailed isoform characterization.

## Discussion

In this study, we present a three-generation family with six individuals showing a striking novel phenotype, characterized by a furrowed or cleft tongue, a narrow face, reddish hair, freckles and various foot deformities. Despite intensive research, we were not able to identify a similar case in the literature and to make a specific clinical diagnosis, so we decided to perform variant analysis by whole-exome sequencing. We selected the three most distantly related affected family members to reduce the amount of shared benign variants and to maximize the segregation filtering power. Focusing on heterozygous variants consistent with a dominant transmission in line with the family history, we identified a heterozygous variant, c.3G>T, in *TP63* affecting the translation initiation codon (p.1Met?). Heterozygous variants in *TP63* have been linked to several syndromic and isolated diseases [5, 18] with partly overlapping phenotypes and various combinations of the following features: ectodermal dysplasia (e.g., nail dysplasia, sparse hair, hypopigmentation, freckling, tooth abnormalities), split-hand/foot malformation/syndactyly, lacrimal duct obstruction, hypoplastic breasts and/or nipples, ankyloblepharon filiforme adnatum, hypospadias and cleft lip/palate [1, 2]. The large number of p63 isoforms might explain the wide spectrum of *TP63*-associated diseases (Fig. 1). Two different transcription start sites lead to two main classes of transcripts: TAp63 and ΔNp63 [6, 8, 9]. Furthermore, alternative splicing at the 3ʹend of both the TAp63 and ΔNp63 transcripts generates at least three different C-terminal variants (α, β, and γ) [8, 10]. Cleft tongue has not been linked as a characteristic feature to *TP63*-related disorders until now [21–23]. However, other *TP63* variants have been described to cause forms of orofacial clefts including cleft lip, cleft palate and cleft uvula [24–26]. Interestingly, especially loss-of-function variants in *TP63* seem to result in a phenotype dominated by oral clefting [5]. So far, no *TP63* variant has been described that affects the translation initiation codon of the first transcription start site, neither as disease-causing (HGMD Database Professional 2020.4) nor in healthy controls (https://gnomad.broadinstitute.org; last access date 10/02/2021, [20]). The most likely used next start codon is at position 40, leading to a shortened version of the TAp63 isoforms. For further verification, we validated the WES data and performed segregation analysis. The *TP63* variant, c.3G>T, was found in all six affected family members by Sanger sequencing, supporting our theory that this unique variant causes a novel *TP63*-associated phenotype.

The specific phenotype of the reported patients might indicate that the affected TAp63 isoforms are especially expressed during a specific period of the development during embryogenesis. Tongue development is a complex process starting around week 4 of embryonic development [27]. The muscles of the tongue predominantly derive from the myoblasts, which originate in the occipital somites, and tongue connective tissue and vasculature are derived from cranial neural crest cells [28, 29]. Reciprocal interactions between cranial neural crest cells and myogenic cells are essential in the coordination of tongue development, and deep single median furrows on the dorsal surface of the tongue or a cleft tongue can occur if this complex process is disturbed [30]. Expression analysis of different *TP63* isoforms indicates that TAp63 is the dominant isoform expressed in skeletal muscle and plays a critical role specifically in late myogenic differentiation stages [31], providing an explanation for the observed phenotypic differences between patients described in this study and previously described TP63-associated phenotypes. Specifically, TAp63γ contributes to muscle growth, remodeling and functional differentiation by controlling specific sub-sets of target genes, and it is involved in the formation of atrophic myotubes and reduced myoblasts fusion index [32]. Dysfunction of TAp63, therefore, might directly affect myoblast fusion during tongue development, resulting in a deep-furrowed/cleft tongue, and, furthermore, provide an explanation for the muscular hypotonia that is present in varying degrees in all our patients. Further investigations are required to verify this hypothesis.

In general, the occurrence of a cleft tongue with or without other abnormalities is rare. Most of the other syndromes related with cleft/furrowed or bifid tongue, such as orofaciodigital syndrome 1 [MIM 311200], Robinow syndrome [MIM 180700] or distal arthrogryposis type 5D [MIM 615065], are as well associated with other forms of orofacial clefts, e.g., cleft lip and/or cleft palate. Among the genetic causes associated with these phenotypes, pathogenic variants in *DVL1/DVL3* and *WNT5A* (all (i.a.) for Robinow syndrome) and *TCTN3* (orofaciodigital syndrome 4) have been identified. Encoded proteins are involved in different cellular pathways which, like the sonic hedgehog (SHH) and WNT pathways, are key signaling pathways during development and maintenance of various tissues and, similar to p63, regulate expression of a huge variety of target genes [33–35]. Interestingly, it has been shown that p63 can modulate both pathways and act as an activator of SHH signaling as well as a repressor of canonical WNT signaling in different tissues and developmental stages [36, 37]. However, these regulations can be exerted by different isoforms of *TP63*, including ΔNp63 variants, which are not affected by the variant identified in this study [38]. Therefore, it remains highly speculative whether the observed phenotype is caused by direct interaction of p63 with these pathways.

Additionally, we found by extensive research of the literature a further hint in line with our assumption. Pries et al. reported in 1974 three patients with EEC syndrome showing a furrow in the midline of the tongue [39], too. Consistent with the clinical diagnosis of EEC syndrome, these patients showed further malformations including ectodermal dysplasia as well as cleft lip and palate. One of the patients with furrowed tongue reported by Pries et al. had additional split-hand/foot malformation. Overall, they were more severely affected than the patients described here. The genetic cause of these cases is unfortunately unknown. We can only speculate what kind of variant might have caused their specific phenotype, but still, the phenotypic overlap to the patients presented in this study is quite striking. Interestingly, muscular hypotonia was not mentioned as a clinical feature in these three cases. Actually, it remains unclear whether the muscular hypotonia that is present in varying degrees in all of our patients can be explained by the *TP63* variant. Despite intensive efforts, we were not able to identify any additional genetic cause of this. Presenting these cases will contribute to identify further families with hereditary cleft tongue due to variants in *TP63*, which will help to gain further insights into p63-associated pathomechanisms and to answer these open questions.

In summary, our findings indicate that heterozygous variants in *TP63* affecting the first translation initiation codon result in a novel phenotype dominated by a cleft tongue, expanding the complex genotypic and phenotypic spectrum of *TP63*-associated disorders.

## Data Availability

The data that support the findings of this study are available on request from the corresponding author. The data are not publicly available due to privacy or ethical restrictions.
